# Of the Induction of Premature Labor, in the Obstinate Vomiting of Pregnant Women

**Published:** 1853-09

**Authors:** 


*Of the induction of Premature Labor, in the Obstinate Vomiting of Pregnant Women.* By M. Cazeaux. (At a late meeting of the Société de Médecine de Paris.)

M. Cazeaux referred to a case of obstinate vomiting which appeared to yield under the employment of the extract of belladonna, applied to the neck of the uterus, which he had reported to the society some months ago. A short time since, he had had occasion to observe a similar but less fortunate case. M. Trousseau had called him to attend a young woman, two months and a half advanced in pregnancy, who literally vomited everything which she took into her stomach. She suffered from epigastric spasms with complete privation of sleep, with fever and emaciation. Nevertheless the appearance of the woman was tolerably good, and M. Cazeaux tried the belladonna, but without the least benefit. M. Trousseau then called in consultation MM. Dubois, Monnier, Andral and Cazeaux. The first day it was determined, on the proposition of M. Andral, to employ opium. The medicine was without effect. M. Cazeaux added that it augmented the vomiting. It then occurred to M. Trousseau to administer pills of meat, which he had prepared by Mialhe. The patient continued to vomit, however, though she retained ten or twelve of the pills.

At the second consultation abortion was proposed. M. Cazeaux alone opposed this proposition. Notwithstanding his objections, abortion was decided on; he retired.

The operation was attempted by M. Paul Dubois, and did not succeed for three days. After the abortion the vomiting ceased, but the fever persisted, and the other phenomena augmented. In a few days rheumatismal pains occurred in the shoulders, and afterward numerous abscesses in the same region. In short, the woman died fifteen days after the abortion. No autopsy was allowed.

M. Cazeaux considered the abscesses the result of a uterine phlebitis. He remarked that this was a seventh case of ill-success to be added to those already known to science. Hitherto there has been only one fortunate case in eight. He concluded, therefore, that the expectant plan was preferable to an operation.

M. Jacquemin had lately observed a case of obstinate vomiting in a young primiparous woman, four months advanced in pregnancy, residing in Havre. The physicians of that locality tried ineffectually the means recommended in such cases. The patient came to Paris and was placed under the care of M. Jacquemin. He employed without success, enema of laudanum, and blisters dressed with morphia to the epigastrium. She was in a very grave condition: besides the vomiting, the intellectual faculties were perverted. M. Jacquemin thought of proposing abortion, but he wished first to have the opinion of M. Moreau. As this young woman was in the habit of drinking brandy and cologne water, the consultation decided to try first champagne wine; the patient drank three glasses of it daily; the vomiting ceased from that moment.

Like M. Cazeaux, M. Jacquemin had observed that opium augmented the vomiting.

M. Cazeaux observed that this case was the more interesting because the patient of M. Jacquemin had arrived at the third stage of that affection which, according to an aphorism, inevitably involves the death of the patient.

M. Rayer has been understood to say that he has cured two cases of obstinate vomiting by sherry wine. It is certain that the preparations of alcohol have succeeded in these cases beyond all hope.

M. Loir had observed a case similar to those of MM. Cazeaux and Jacquemin. The antiphlogistic method was used, and the patient succumbed at the end of two months.—*Revue Médicale de Cayol*, from *Virginia Med. and Surg. Journal.*

